# Melatonin associated with bacterial cellulose-based hydrogel improves healing of skin wounds in diabetic rats

**DOI:** 10.1590/acb399024

**Published:** 2024-10-25

**Authors:** Jaiurte Gomes Martins da Silva, Ismaela Maria Ferreira de Melo, Érique Ricardo Alves, Glícia Maria de Oliveira, Anderson Arnaldo da Silva, Flávia Cristina Morone Pinto, José Lamartine de Andrade Aguiar, Diego Neves Araújo, Valéria Wanderley Teixeira, Álvaro Aguiar Coelho Teixeira

**Affiliations:** 1 Universidade Federal Rural de Pernambuco – Department of Animal Morphology and Physiology – Graduate Program in Animal Bioscience – Recife (PE) – Brazil.; 2 Universidade Federal de Alagoas – Maceió (AL) – Brazil.; 3 Universidade Federal de Pernambuco – Department of Biochemistry – Graduate Program in Therapeutic Innovation – Recife (PE) – Brazil.; 4 Universidade Federal de Pernambuco – Graduate Program in Biosciences and Biotechnology in Health – Recife (PE) – Brazil.; 5 Universidade Federal de Pernambuco – Department of Surgery – Graduate Program in Surgery – Recife (PE) – Brazil.

**Keywords:** Biopolymers, Diabetes Mellitus, Bandages, Melatonin, Histology

## Abstract

**Purpose:**

To describe the effects of melatonin associated with bacterial cellulose-based hydrogel on healing of skin wounds in diabetic rats.

**Methods:**

Streptozotocin was used to induce diabetes in Wistar rats. After wound induction, animals were randomly divided into groups GC, GDCC, GDCB, and GDMCB. Animals were evaluated in days 3, 7, and 14 for the following variables: glycemic levels, histopathological and histochemical analyses, healing rate, morphometry and C-reactive protein.

**Results:**

There was no change in glycemic levels in the diabetic animals as a result of the treatments; histopathological analyses showed better healing in GDCB and GDMCB groups, as well as histochemistry; at day 14, the highest healing rate was observed in animals from the GDMCB group, reaching almost 100%; morphometry revealed a significant increase of fibroblasts and reduction of macrophages and blood vessels in lesions treated with bacterial cellulose associated or not with melatonin when compared to the other experimental groups. There was also an increase in C-reactive protein in GDCC group at day 14.

**Conclusion:**

Bacterial cellulose-based dressings associated with systemic melatonin showed beneficial results in experimentally induced wounds in diabetic rats, favoring the healing process.

## Introduction

Diabetes mellitus (DM) is a chronic condition consisting of a group of metabolic disorders caused by a hyperglycemic state in the body, thus affecting all systems of an individual. It is estimated that more than 537 million people live with diabetes worldwide and that one in 10 have the disease^1^^,^ 2.

Diabetic individuals have a deficiency in wound healing, as there is a compromised blood perfusion, hindering an adequate supply of oxygen and nutrients^3^. In addition, chronic wounds are a health problem that has devastating consequences for patients and contributes to great costs for health systems and societies, in which the cost of hospitalization can range from US$ 12,851 to US$ 16,267^4^.

Conventional alternatives such as debridement, and specialized dressings, such as electroactive nanofibers^5^, plasma-based treatment^6^, and chitosan hydrogel^7^, have been used to improve healing in patients who have impaired healing. However, they may have disadvantages due to their high cost, difficult handling and poor anti-inflammatory and antibacterial properties.

In the last decade, studies have shown that bacterial cellulose-based biomaterials have been used as healing agents in hard-to-heal wounds, as they are non-toxic, biocompatible and inexpensive^8^^–^ 11. Bacterial cellulose (BC) is one of these promising classes of biopolymers, since it can control wound exudates, resulting in better healing^12^^–^ 14, and may be presented in different ways, such as gel, hydrogel, dressings, foams, and perforated membrane^15^^–^ 17.

Melatonin, a hormone produced by the pineal gland, has shown a positive effect on wound healing in topical^18^^–^ 20 or systemic application^21^^–^ 24. However, some studies have investigated the association of this hormone with some biomaterials such as chitosan and lectin^25^ in order to accelerate healing, thereby seeking to generate new therapeutic options. Thus, aiming to expand the range of these options, we evaluated the effect of systemic administration of melatonin associated with a bacterial cellulose-based hydrogel dressing on the healing of cutaneous wounds in diabetic rats.

## Methods

This study followed the principles established by the Brazilian laws on the use and creation of animals (Law no. 11,794/2008), which regulate research with animals in Brazil, and it was approved by the Ethics Committee in the Use of Animals of the Universidade Federal Rural de Pernambuco (UFRPE), under license nº 052/2019.

Forty-eight albino rats (*Rattus norvegicus albinus*) of the Wistar lineage, 60 days old and weighing approximately 250 ± 30 g, from the vivarium of the Department of Animal Morphology and Physiology of the UFRPE were used. The animals were maintained in an environment with controlled temperature (22 ± 1ºC) and photoperiod (12 h light and 12 h dark) and were fed and watered ad libitum.

### Experimental design and anesthetic procedures

The animals were evaluated daily for clinical and wound characteristics. Animals were randomly divided into four groups, each with 12 animals with skin lesions, as shown below:

- GC: non-diabetic rats;
- GDCC: diabetic rats treated with amorphous commercial healing agent with alginate;
- GDCB: diabetic rats treated with BC;
- GDMCB: diabetic rats treated with melatonin and BC.

Three animals from each group were euthanized in days 3, 7 and 14.

### Diabetes induction

Diabetes was induced by intraperitoneal administration of a streptozotocin (STZ) solution (Sigma Chemical Co., United States of America) after a 14-hour fasting period and confirmed on the fifth day after application. STZ was diluted in sodium citrate buffer at 10 mM and pH 4.5, in a single dose of 60 mg/kg of animal weight. Non-diabetic animals (GC) received similar doses of saline solution, and, 30 minutes after administration, all animals were fed normally. Only animals that had blood glucose above 200 mg/dL (confirmed by the Accu-Chek Activ Glucometer Kit, five days after induction) were included in the study, except for the non-diabetic group (GC). All treatments started on the day diabetes was confirmed on the animals.

### Excisional wound model

The animals were anesthetized with ketamine hydrochloride (80 mg/kg) and xylazine (6 mg/kg), intramuscularly. Wounds were made on the back of each animal using a 20-mm dermatological punch. For this, a 6-cm long and 4-cm wide trichotomy was performed, after which the skin fragment was excised, in the center of the epilated area, until the muscular fascia was exposed. Anesthetic level was confirmed by testing the caudal reflex, foot reflex and vibrissae movement.

### Melatonin treatment

Melatonin, N-acetyl-5-methoxytryptamine (Sigma Chemical Co., St. Louis, United States of America), was administered in daily injections, intraperitoneally at 6 to 7 p.m., at 10 mg/kg. It was dissolved in 0.2 mL of ethanol and diluted in 0.9 mL of 0.9% NaCl.

### Treatment with bacterial cellulose and commercial healing

BC hydrogel and commercial healing agent (CC) were applied directly to the animal’s wound, once a day, with approximately 0.4 mL. The commercial healing agent used was an amorphous hydrogel with alginate, chosen because of its wide use and similar physicochemical characteristics with BC hydrogel. The BC hydrogel was produced from sugarcane molasses at the Sugarcane Experimental Station of the UFRPE, supplied by the company Polisa Biopolímeros para Saúde Ltda. BC is produced by propagating *Gluconacetobacter hansenii* in a sterile culture obtained from sugarcane molasses. The polymeric mass is mechanically processed to obtain the gel and, subsequently, the hydrogel. All stages of production were developed in accordance with the standard operating procedure, a process developed and patented by Polisa Biopolímeros para Saúde Ltda. Samples of BC hydrogel dressings tested in this study were prepared from a 0.7% concentration.

### Healing rate

After the anesthetic procedure, the wounds were photographed using a digital camera. Then, images were transferred to the computer to be analyzed by the ImageJ software. Healing rate was calculated by comparing the area of the unhealed wound (Arean) with the area of the original wound (Areai), according to Eq. 1^26^:


$$\text{Healing rate}(\%)=1-\left\{\left[{\text{Area}}_{1}-{\text{Area}}_{n}\right]/\text{Area}\right\}\times 100$$ (1)

### Histopathological evaluation

The wounds were analyzed at the end of each experimental day (days 3, 7 and 14). Then, the skin fragments were collected, and samples fixed in 10% buffered formalin for 24 hours. After that, samples were immersed in ethanol solutions with ascending degrees of 50 to 100% each, for 45 minutes to 1 hour, and then three times in xylene. Henceforth, the samples were embedded in paraffin, and 4-mm thick fragments were prepared. The fragments were stained with hematoxylin and eosin for histopathology, and Masson’s trichrome was used for histochemistry. The slides of days 3, 7 and 14 were blindly evaluated by a histopathologist for the presence or absence of inflammatory infiltrate, granulation tissue and blood vessels.

### Histochemical evaluation

Histochemical analysis was performed using Masson’s trichrome staining, in which the result of collagen quantification was expressed by pixel quantification. For this, three slides were used per rat/group. On each slide, four fields were photographed in a zigzag direction, per slide. The images were captured using a Sony video camera, coupled to the Olympus Bx50 microscope, which were submitted to the Gimp 2.0 program, for quantification using the red-green-blue histogram^27^^,^ 28.

### Morphometric evaluation

For the morphometric evaluation, an Olympus Bx 50 optical microscope was used, adapted with a 10x histometric eyepiece (Karl Zeiss Jena model GF – P) equipped with a 100-point reticle. The objective used was 100x. Three slides/rat/group were used. In each slide, five fields were observed, two fields close to the wound/normal skin transition region, on opposite sides, a central field of fragments and two more fields from the wound/normal skin transition region, on opposite sides of the other fragment. The number of fibroblasts (they have a large, elliptical nucleus, little condensed chromatin, and several nuclei), macrophages and blood vessels were counted^29^.

### C-reactive protein levels

C-reactive protein (CRP) was measured in plasma using commercial Labtest kits with a minimum detectable CRP concentration of 0.35 ng/mL and 10% inter-assay coefficient of variation. Sample absorbances were read in duplicate at 520 nm, with wavelength correction at 560 nm outside a standard curve and expressed in micrograms per milliliter^30^^,^ 31.

### Statistical analysis

Normality was tested by Shapiro–Wilk’s test. Based on the results, the data were submitted to analysis of variance. When significant, it was complemented by the Tukey’s and Kramer multiple comparisons test. A significance level of 0.05 (*p* < 0.05) was adopted.

## Results

### Glycemic levels

**Figure 1:**
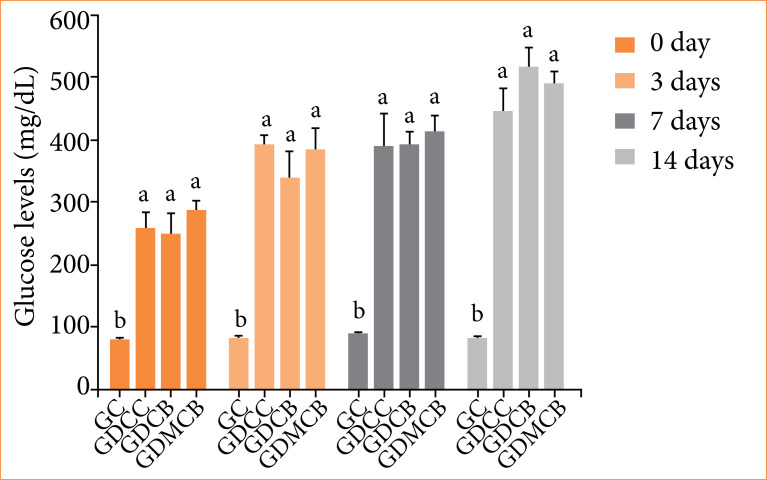
Mean + standard deviation of glycemic levels in animals in experimental groups. Means followed by the same letters do not differ significantly by Tukey and Kramer’s multiple test (*p* < 0.05).

GC: non-diabetic rats; GDCC: diabetic rats treated with amorphous commercial healing agent with alginate; GDCB: diabetic rats treated with bacterial cellulose; GDMCB: diabetic rats treated with melatonin and bacterial cellulose. Source: Elaborated by the authors.

There was no change in the glycemic levels of the groups of diabetic animals due to the treatments and periods considered, but all differed from the CG group (*p* = 0.0012) (Fig. 1). There were no losses of animals during the experimental period and no complications, including infection, related to the procedures.

### Wound healing rate

**Figure 2:**
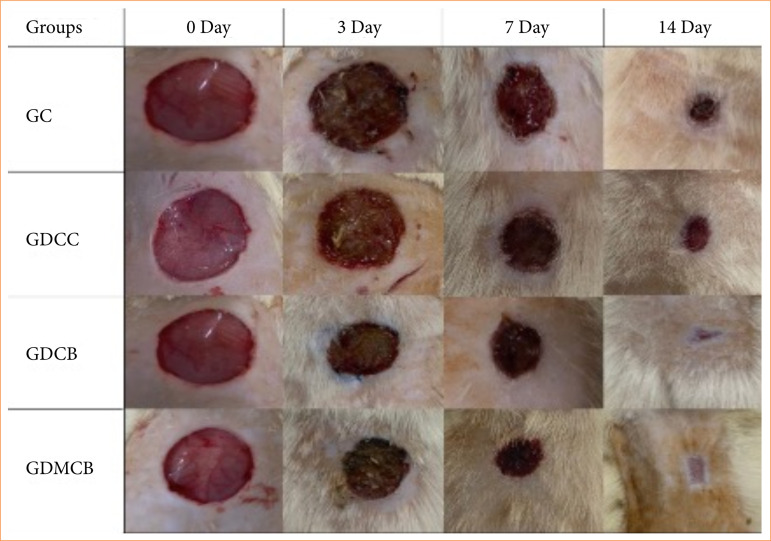
Photograph of the evolution of the cutaneous lesions in the animals of the experimental groups.

GC: non-diabetic rats; GDCC: diabetic rats treated with amorphous commercial healing agent with alginate; GDCB: diabetic rats treated with bacterial cellulose; GDMCB: diabetic rats treated with melatonin and bacterial cellulose. Source: Elaborated by the authors.

At day 3, wounds from the animals groups showed signs of healing characterized by a slight reduction in diameter. At day 7, the animals in the GDCB and GDMCB groups showed more advanced signs of healing compared to the other experimental groups. This behavior was more expressive on day 14, showing a complete healing process (p = 0.0143) (Fig. 2).

**Figure 3:**
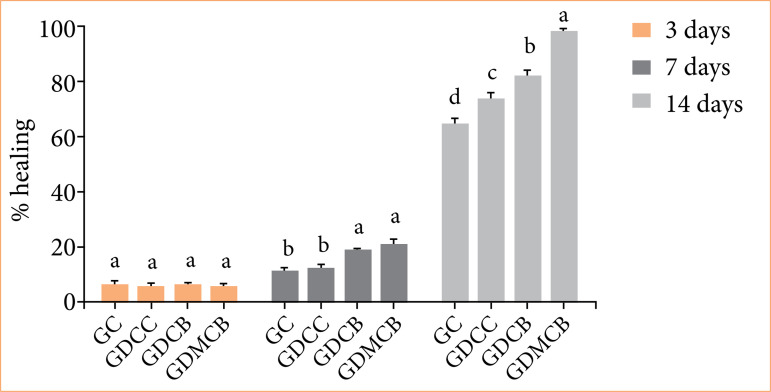
Mean + standard deviation of percentage healing rate of lesions in animals in experimental groups. Means followed by the same letters in the columns do not differ significantly by Tukey and Kramer’s multiple test (*p* < 0.05).

GC: non-diabetic rats; GDCC: diabetic rats treated with amorphous commercial healing agent with alginate; GDCB: diabetic rats treated with bacterial cellulose; GDMCB: diabetic rats treated with melatonin and bacterial cellulose. Source: Elaborated by the authors.

Analysis of healing rate showed no significant differences between treatments at day 3. At day 7, there was an increase in the healing rate in GDCB and GDMCB groups, significantly differing from the other treatments. However, at day 14, the highest healing rate was observed in the animals from the GDMCB group, reaching approximately 100%, differing from the other experimental groups (*p* = 0.0103) (Fig. 3).

### Histopathological analysis

**Figure 4:**
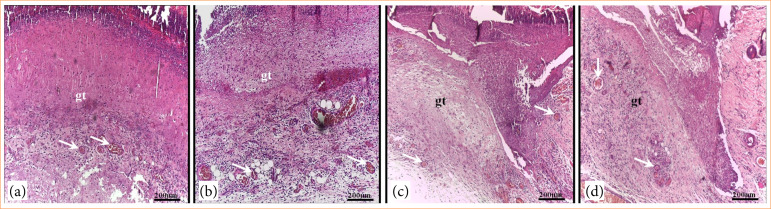
Photomicrograph of skin lesions in animals from experimental groups after three days of induction and respective treatments. **(a)** GC; **(b)** GDCC, **(c)** GDCB, **(d)** GDMCB. Note the presence of granulation tissue (gt) and blood vessels (arrows) in the lesions. Hematoxylin and eosin.

GC: non-diabetic rats; GDCC: diabetic rats treated with amorphous commercial healing agent with alginate; GDCB: diabetic rats treated with bacterial cellulose; GDMCB: diabetic rats treated with melatonin and bacterial cellulose. Source: Elaborated by the authors.

Histopathological analysis revealed that the skin lesions at day 3 had the same characteristics with the presence of an inflammatory infiltrate throughout the granulation tissue and blood vessels. However, these characteristics were less intense in the lesions of GDCB and GDMCB groups (Fig. 4).

**Figure 5:**
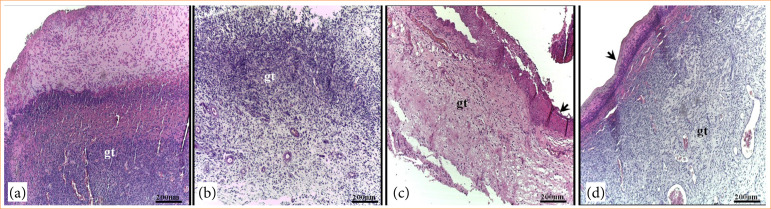
Photomicrograph of skin lesions in animals from experimental groups after seven days of induction and respective treatments. **(a)** GC; **(b)** GDCC, **(c)** GDCB, **(d)** GDMCB. Note the presence of granulation tissue (gt) and re-epithelialization in the lesions (arrowhead). Hematoxylin and eosin.

GC: non-diabetic rats; GDCC: diabetic rats treated with amorphous commercial healing agent with alginate; GDCB: diabetic rats treated with bacterial cellulose; GDMCB: diabetic rats treated with melatonin and bacterial cellulose. Source: Elaborated by the authors.

At day 7, granulation tissue was also observed in GC and GDCC groups. However, in animals from GDCB and GDMCB groups, the lesions showed a re-epithelialization process and a significant reduction in granulation tissue (Fig. 5).

**Figure 6:**
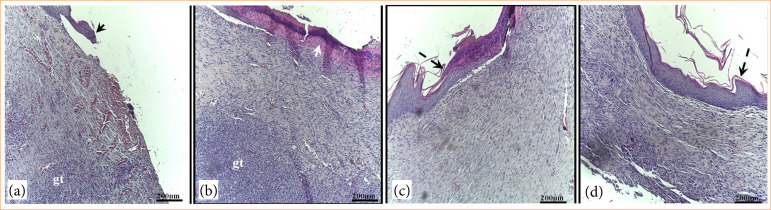
Photomicrograph of skin lesions in animals from experimental groups after 14 days of induction and respective treatments. **(a)** GC; **(b)** GDCC, **(c)** GDCB, **(d)** GDMCB. Note in the lesions the presence of granulation tissue (gt) only in a and b, re-epithelialization (arrowhead) and epithelium in the process of keratinization (dashed arrow). Hematoxylin and eosin.

GC: non-diabetic rats; GDCC: diabetic rats treated with amorphous commercial healing agent with alginate; GDCB: diabetic rats treated with bacterial cellulose; GDMCB: diabetic rats treated with melatonin and bacterial cellulose. Source: Elaborated by the authors.

At day 14, re-epithelialization and the presence of granulation tissue were observed in GC and GDCC groups. In GDCC and GDMCB groups, a process of epithelial keratinization and absence of granulation tissue were observed (Fig. 6).

### Histochemical analysis

**Figure 7:**
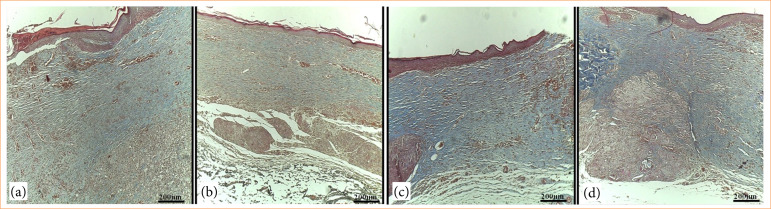
Masson’s trichrome histochemistry in skin lesions evaluated 14 days after induction and respective treatments. **(a)** GC; **(b)** GDCC, **(c)** GDCB, **(d)** GDMCB. Note positive labeling in all groups.

GC: non-diabetic rats; GDCC: diabetic rats treated with amorphous commercial healing agent with alginate; GDCB: diabetic rats treated with bacterial cellulose; GDMCB: diabetic rats treated with melatonin and bacterial cellulose. Source: Elaborated by the authors.

**Figure 8:**
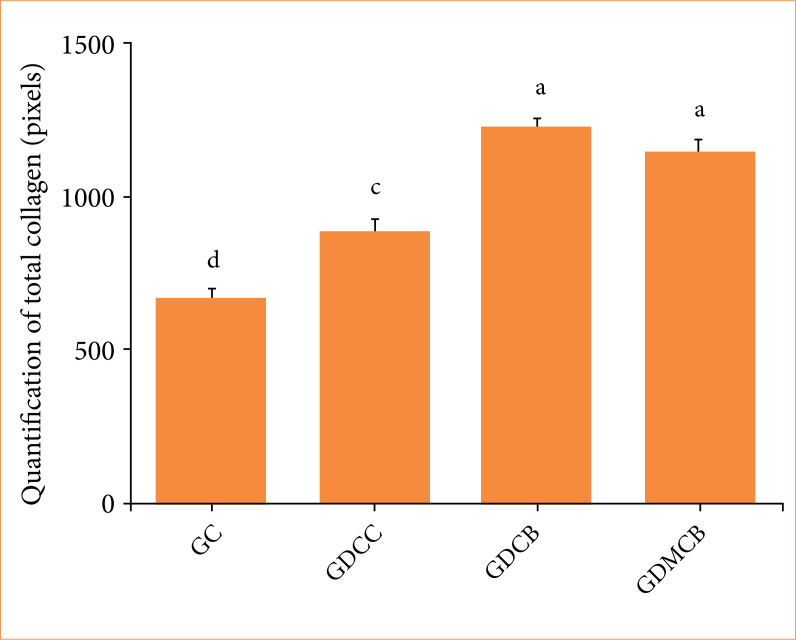
Mean + standard deviation of total collagen quantification in pixels in the skin lesions of animals in the experimental groups. Check a significant increase in GDCB and GDMCB groups. Means followed by the same letters in the columns do not differ significantly by Tukey and Kramer’s multiple test (*p* < 0.05).

GC: non-diabetic rats; GDCC: diabetic rats treated with amorphous commercial healing agent with alginate; GDCB: diabetic rats treated with bacterial cellulose; GDMCB: diabetic rats treated with melatonin and bacterial cellulose. Source: Elaborated by the authors.

Masson’s trichrome histochemistry for total collagen in the lesions at day 14 showed positive staining in all lesions (Fig. 7). However, the quantification in pixels showed a lower collagen content in the lesions of the animals in GC and GDCC groups compared to the other experimental groups (*p* = 0.0034) (Fig. 8).

### Morphometric analysis

**Figure 9:**
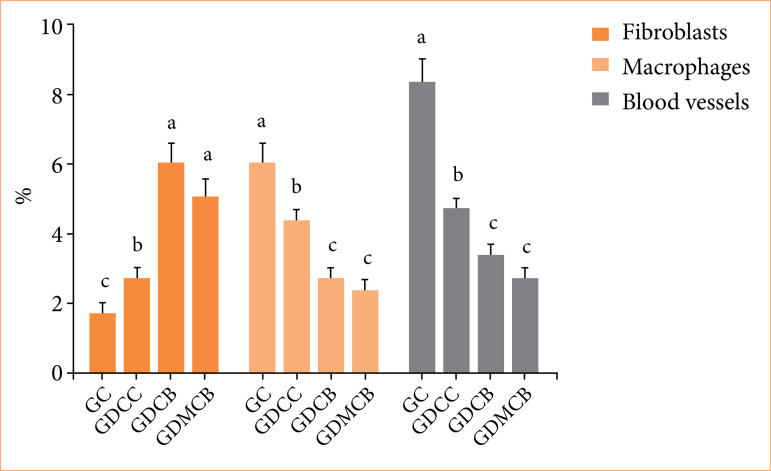
Mean + standard deviation of the percentage of fibroblasts, macrophages and blood vessels in the skin lesions of the animals in the experimental groups at day 14. Means followed by the same letter do not differ significantly by Tukey and Kramer’s multiple comparisons test (*p* > 0.05).

GC: non-diabetic rats; GDCC: diabetic rats treated with amorphous commercial healing agent with alginate; GDCB: diabetic rats treated with bacterial cellulose; GDMCB: diabetic rats treated with melatonin and bacterial cellulose. Source: Elaborated by the authors.

Morphometrics revealed that lesions treated with BC, associated or not with melatonin, showed a significant increase in fibroblasts and a reduction in macrophages and blood vessels, when compared to lesions in animals from the other experimental groups, which had fewer fibroblasts and a large number of macrophages and blood vessels (0.0087) (Fig. 9).

### Plasma C-reactive protein levels

**Figure 10:**
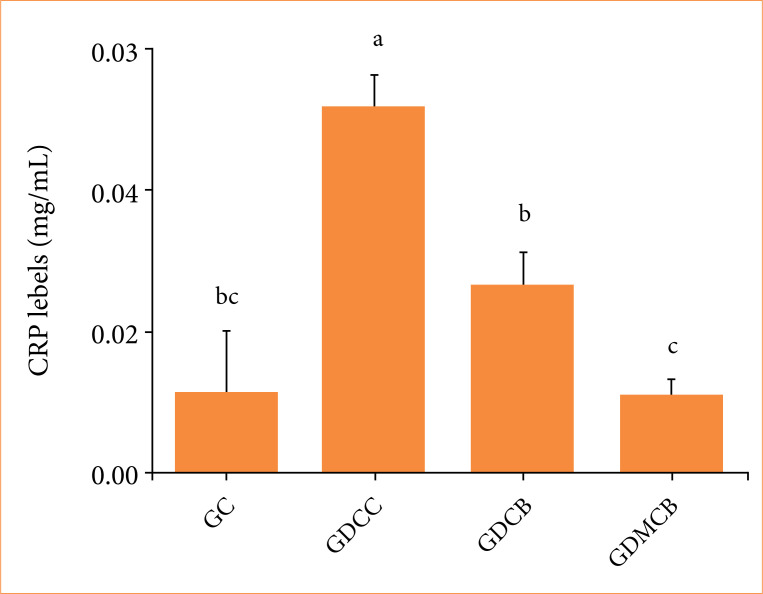
Mean + standard deviation of CRP levels in animals from experimental groups at day 14. Means followed by the same letter do not differ significantly by Tukey and Kramer’s multiple comparisons test (*p* > 0.05).

GC: non-diabetic rats; GDCC: diabetic rats treated with amorphous commercial healing agent with alginate; GDCB: diabetic rats treated with bacterial cellulose; GDMCB: diabetic rats treated with melatonin and bacterial cellulose; CRP: C-reactive protein. Source: Elaborated by the authors.

Plasma analysis of CRP showed that, at day 14, there was an increase in its concentration in the GDCC group, and the lowest levels in GDCB, GC and GDMCB groups, respectively (0.0174) (Fig. 10).

## Discussion

Common biomass materials for dressings include chitosan, gelatin, hyaluronic acid, etc.^32^^–^ 34. These materials have good biocompatibility, degradability, mechanical properties, etc., but BC has advantages in wound dressings because of its characteristics of high tensile strength, good flexibility, strong water holding capacity, significant gas permeability and liquids, great compatibility with living tissues^35^. In this study, we showed the effects of the association of systemic melatonin application and topical use of BC hydrogel on wound healing in diabetic rats.

The increase in serum glucose levels can lead to structural and functional changes in the target organs of diabetic patients, including the skin^36^^,^ 37. In this study, blood glucose levels of STZ-induced diabetic rats were significantly higher compared to GC group, but there was no difference for GDCC, GDCB, and GDMCB groups. These higher blood glucose levels were observed throughout the study period and are caused by the effects of streptozotocin on pancreatic β cells. STZ leads to the development of insufficient insulin production and, consequently, to the elevation of the blood glucose level^38^.

Studies report that the administration of melatonin (10mg/kg), for approximately 16 days, in diabetic animals, protects the pancreas and improves glucose homeostasis and attenuates the effects of STZ. However, there are reports showing no difference between groups treated with melatonin^39^^–^ 41. The lack of changes in blood glucose levels in the melatonin group could be related to the melatonin dosage, or the route of melatonin administration used in the study.

Regarding healing rate, at day 7, the groups treated with BC and melatonin + BC showed significantly higher rates than the control group and the one with a commercial healing agent. However, at day 14, only the associated treatment proved to be more effective with the highest healing rate. Similar studies showed melatonin-loaded injectable hydrogel induced granulation tissue formation and accelerated wound healing^42^, demonstrating that melatonin regulates the release of inflammatory mediators. Research also indicates that this hormone acts in the initial inflammatory phase, activating mediators such as phospholipase A2 and arachidonate 5-lipoxygenase^43^^,^ 44.

Regarding the effects of BC hydrogel dressing on healing rate, a recent study macroscopically demonstrated faster closure of the diabetic wound, close to 99%^45^. Our results showed that wound closure reached 98% in animals from GDMCB group at day 14. In addition, in a histopathological evaluation of wounds, the presence of inflammatory infiltrate, granulation tissue and blood vessels are common characteristics, especially in the initial phase of inflammation. We verified that the animals in GDCB and GDMCB groups presented an inflammatory phase with less intensity and duration; already at day 7, they showed signs of re-epithelialization. However, the high healing rate observed in the association of BC hydrogel with melatonin suggests a synergistic potentiating effect of the healing process.

Healing studies with BC have shown that there is no inhibitory effect on various cell types including fibroblasts and keratinocytes^45^^–^ 48, in which the migration of these cells is a crucial step in accelerating the healing process. It is believed that fibroblasts are the first cells to migrate to the wound site along with endothelial cells, and by keratinocytes that induce re-epithelialization. Subsequently, a remodeling stage occurs involving a second migration of fibroblasts^49^^,^ 50. In addition to this, we should mention that melatonin can act as a paracrine or autocrine regulator in leukocyte communication in wound healing^43^. Therefore, this could explain the rapid evolution of healing in animals from GDCB and GDMCB groups.

Total collagen quantification in lesions of the animals in the experimental groups showed a significant increase in GDCB and GDMCB groups compared to GC and GDCC groups, with a greater production in the GDCB group, even without significance. A recent study showed that application of melatonin accelerates the maturation of skin wounds and increases collagen deposition. This study concluded that melatonin favors early maturation of collagen fibers, greater collagen deposition, and faster formation of scarring connective tissues^25^. Studies show that BC dressings promote collagen production, that BC is biocompatible, favoring cell migration, promoting a favorable environment for healing^14^^,^ 51 ^,^ 52. These findings are important, as collagen metabolism directly affects the quality of wound repair.

Morphometry revealed an increase of fibroblasts at day 14 in GDCB and GDMCB groups compared to GC and GDCC groups, with a bigger production in the GDCB group, even without significance. BC produces an ideal microenvironment for the synthesis of fibroblasts, and consequently for collagen, as studies showed^11^^,^ 15 ^,^ 53 ^,^ 54. These same effects, however, in studies with melatonin are also reported^25^^,^ 55. The morphometry of macrophages and blood vessels showed an increase at day 14 in GC and GDCC groups, which may indicate a delay in healing compared to the other groups. Observing blood vessels, inflammatory cells and fibroblasts are important, because, if cellularity persists in the area, the formation of hypertrophic scars or keloids may occur.

CRP exhibits heightened expression during inflammatory conditions^56^. At the end of our study, it was possible to observe an increase in CRP levels in the GDCC group and a decrease in GC, GDCB, and GDMCB groups. Elevated glucose levels can trigger microvascular changes and increased production of inflammatory factors, including CRP^57^. Studies reinforce that melatonin in patients with diabetes results in a significant decrease in serum CRP levels^58^, or it may not change them^59^.

## Conclusion

Regarding the results obtained in the experimental model of skin wounds in diabetic rats, it was possible to conclude that the application of BC-based dressings, associated to melatonin, promoted faster wound closure, increased the production of fibroblasts and collagen fiber deposition and attenuated the inflammatory effects, providing better healing.

## Data Availability

All data sets were generated or analyzed in the current study.
